# Directed evolution of a three-finger neurotoxin by using cDNA display yields antagonists as well as agonists of interleukin-6 receptor signaling

**DOI:** 10.1186/1756-6606-4-2

**Published:** 2011-01-07

**Authors:** Mohammed Naimuddin, Suzuko Kobayashi, Chihiro Tsutsui, Masayuki Machida, Naoto Nemoto, Takafumi Sakai, Tai Kubo

**Affiliations:** 1 Applied Gene Technology, Institute for Biological Resources and Functions, National Institute of Advanced Industrial Science and Technology, Central 6, 1-1-1, Higashi, Tsukuba, Ibaraki 305-8566, Japan; 2 Innovation Center for Start-ups, National Institute of Advanced Industrial Science and Technology, 2-2-2, Marunouchi, Chiyoda-ku, Tokyo, 100-0005, Japan; 3 Molecular Neurophysiology, Neuroscience Research Institute, National Institute of Advanced Industrial Science and Technology, Central 6, 1-1-1 Higashi, Tsukuba, Ibaraki 305-8566, Japan; 4 Department of Regulation Biology, Faculty of Science, Saitama University, 255 Shimo-okubo, Sakura-ku, Saitama, 338-8570, Japan; 5 Janusys Corporation, #508, Saitama Industrial Technology Center, Skip City, 3-12-18 Kami-Aoki, Kawaguchi, Saitama, 333-0844, Japan; 6 United Graduate School of Drug Discovery and Medical Information Sciences, Gifu University, 1-1 Yanagido, Gifu, Gifu 501-1193, Japan; 7 Biomedical Research Institute, National Institute of Advanced Industrial Science and Technology, Central 6, 1-1-1 Higashi, Tsukuba, Ibaraki 305-8566, Japan

## Abstract

**Background:**

Directed evolution of biomolecules such as DNA, RNA and proteins containing high diversity has emerged as an effective method to obtain molecules for various purposes. In the recent past, proteins from non-immunoglobulins have attracted attention as they mimic antibodies with respect to binding potential and provide further potential advantages. In this regard, we have attempted to explore a three-finger neurotoxin protein (3F). 3F proteins are small (~7 kDa), structurally well defined, thermally stable and resistant to proteolysis that presents them as promising candidates for directed evolution.

**Results:**

We have engineered a snake α-neurotoxin that belongs to the 3F family by randomizing the residues in the loops involved in binding with acetylcholine receptors and employing cDNA display to obtain modulators of interleukin-6 receptor (IL-6R). Selected candidates were highly specific for IL-6R with dissociation constants and IC50s in the nanomolar range. Antagonists as well as agonists were identified in an IL-6 dependent cell proliferation assay. Size minimization yielded peptides of about one-third the molecular mass of the original proteins, without significant loss of activities and, additionally, lead to the identification of the loops responsible for function.

**Conclusions:**

This study shows 3F protein is amenable to introduce amino acid changes in the loops that enable preparation of a high diversity library that can be utilized to obtain ligands against macromolecules. We believe this is the first report of protein engineering to convert a neurotoxin to receptor ligands other than the parent receptor, the identification of an agonist from non-immunoglobulin proteins, the construction of peptide mimic of IL-6, and the successful size reduction of a single-chain protein.

## Background

*In vitro *evolution of proteins is an increasingly promising approach for introducing desired, novel changes that can modulate the properties and/or functions of proteins [1]. In this regard, technologies such as phage display, ribosome display, mRNA/cDNA display and others [2-6] that couple the phenotype (expressed proteins) to their genotype (DNA, mRNA or cDNA) have shown considerable promise, allowing proteins with desired functions to be selected from large totally random and scaffold libraries [7].

Protein scaffolds can be either naturally occurring or *de novo *synthesized, and have defined structures that contain amenable regions such as loops that can be engineered to accommodate completely novel properties, in particular binding and inhibition [8,9]. Disulfide-containing scaffolds, such as α-amylase inhibitor (tendamistat), bovine pancreatic trypsin inhibitor (BPTI; Kunitz domain), EETI-II (knottin) and related proteins are attractive due to their small size, defined structures and remarkable stability. These scaffolds have been successfully engineered for various purposes, such as protease inhibitors, although none have been reported to bind or inhibit macromolecules such as receptors [8]. Here, we report the engineering of a three-finger scaffold that provides a benchmark in the development of regulatory proteins that can modulate the function of interleukin 6 receptor (IL-6R).

3F proteins are found in a variety of organisms such as the elapidae snake and mammals, including humans [10-13]. These are small proteins (MW: 7-8 kDa) with 4-5 disulfide bonds, β-structure(s) and three protruding loops that provide the topological basis for the three-finger structure [10,14]. The striking features of the 3F-protein family are strict conservation of cysteine frameworks and high sequence diversity in the loop (corresponding to finger) regions. This may reasonably make 3F toxins in nature with broad spectrum in target molecules, such as ion channels, receptors, proteases, etc [14]. This class of protein exhibits high temperature stability, resistance to proteases and low immunogenicity. The exceptional specificity of 3F proteins for their respective receptors, conferred by the residues on the tips of the loops, has been extensively investigated [15,16]. These properties make 3F proteins excellent candidates for evolutionary engineering aimed at obtaining molecules for research, diagnostics and therapeutics.

We chose IL-6R to obtain modulatory molecules from the 3F snake neurotoxin library. Interleukin-6 (IL-6) is a multifunctional cytokine regulating cell growth, differentiation, and other cellular functions [17]. The activity of IL-6 is exerted through IL-6R [17-19], and IL-6 is known to play a role in the pathogenesis of a variety of diseases such as Rheumatoid arthritis, Castleman's disease and others [18,20,21]. Therefore, molecules regulating IL-6R-mediated signaling of IL-6 are important for research, diagnostics and therapeutics. In this paper, we report the identification of modulators of IL-6R from a 3F library, and demonstrate the potential of the 3F scaffold in generating novel molecules for macromolecular receptors. The approach involved the directed evolution of the 3F protein library using cDNA display [6]. Further, we identified the functional loops by minimizing the size of the scaffold while retaining function comparable to the intact scaffold. We believe this is the first report of the engineering of a three-finger scaffold for a macromolecular receptor, the simultaneous identification of agonist and antagonist molecules of IL-6R, the generation of a peptide mimic of IL-6, and the downstream engineering of the scaffold in order to identify the functional loops.

## Results

### Overview of cDNA display technology

**Figure 1:**
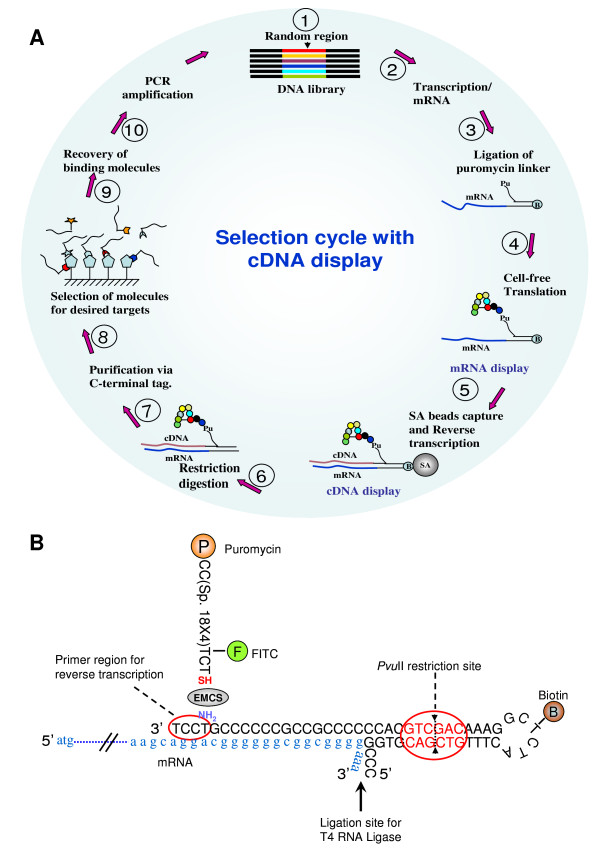
**Overview of cDNA display and construction of the three-finger (3F) scaffold library**. (A) cDNA display is based on the formation of a covalent fusion between the expressed protein (phenotype) and the encoding cDNA (genotype) via a puromycin attached to an oligonucleotide linker. The puromycin linker is central to this technology (see B). In step 1, the library is prepared with the construct to be adopted for the cDNA display (Additional file 1, Fig. S1 and Additional file 2, Table S1 online), transcribed into mRNA and ligated to the puromycin linker in the presence of T4 RNA ligase (steps 2 and 3). The mRNA-linker conjugate is then translated in a cell-free translation system (reticulocyte lysate) and the covalent linkage between the linker and protein is formed in the presence of high salt (step 4). Rapid purification is achieved using biotin-streptavidin. cDNA is then synthesized by reverse transcription in step 5 utilizing the 'built-in' primer of the linker. In step 6, the genotype-phenotype complex is released from the beads by restriction digestion with *Pvu*II. Further, full length proteins are purified via the C-terminal 6xHis (step 7). Following selection of molecules against the immobilized targets, the bound molecules are recovered, purified, amplified (steps 8-10) and used in the next round of selection. (B) Schematic of the Puromycin linker and its functional features. The puromycin linker used in this study consists of four essential functional features. First, a ligation site for mRNA, a biotin moiety for rapid purification using immobilized streptavidin (SA), a primer region for reverse transcription and a restriction site for the release of the complex from the immobilized SA beads.

An overview of the selection process using cDNA display technology is given in Figure 1A. The puromycin linker is central to this technology and facilitates rapid ligation of the mRNA, linking of the expressed protein to its genotype (mRNA), and rapid reverse transcription at relatively low temperature (42°C) (Figure 1B). As a result, unlike related technologies [4,5], the expressed protein is linked to the cDNA (steps 3-5) which provides stability to the complex compared to the unstable mRNA [6]. Furthermore, the stability of cDNA facilitates selection under stringent conditions such as pH, temperature, *etc*.

### Three-finger (3F) library construction and characterization

**Figure 2:**
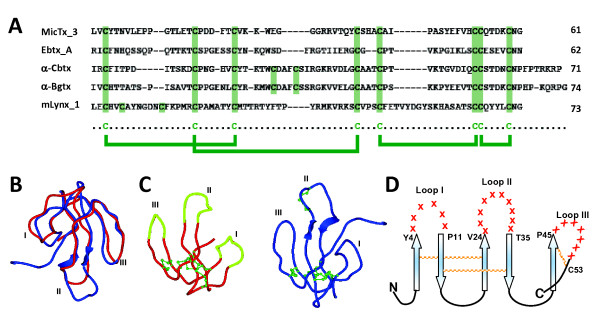
**Sequence and structure comparison of MicTx3 with other three-finger proteins**. (A) Sequence alignment of MicTx3 and representative three-finger proteins. Mature forms of MicTx3, erabutoxin A (Ebtx_A; UniProt Acc#P60775), α-cobratoxin (α-Cbtx; P01391), α-bungarotoxin (α-Bgtx, P60615) and mouse lynx1 (mLynx_1, Q9WVC2) were aligned by Clustal W method using the software, Lasergene ver.7 (DNASTAR, Madison, WI, USA). Conserved cysteine residues among the toxins are highlighted in green, and the disulfide bonds expected from analysis of α-Bgtx are shown by lines. The number of amino acid residues is shown on the right. (B) Superimposition of three-dimensional structures of the MicTx3 model and the α-Bgtx experimental structure. The three-dimensional model of MicTx3 was generated by the software, Internal Coordinate Mechanics (Molsoft, La Jolla, CA, USA), using the α-Bgtx structure (PDBID: 1ik8) as a modeling template. MicTx3 is shown in red and α-Bgtx is in blue. The numbers in Roman numerals represent the loop numbers. (C) Comparison of the MicTx3 model and the α-Bgtx experimental structure shown in (B). MicTx3 is shown in red and α-Bgtx is in blue. Disulfide bonds are colored green. In MicTx3, the loop sections highlighted in fluorescent green show the residues at the tips of the loops that were randomized in order to construct the library. (D) Schematic of the library based on the 3F scaffold. The anti-parallel β-sheets are depicted as blue arrows and the randomized loop residues are indicated as red crosses. Numbers such as Y24 and P11 indicate residues adjacent to the randomized residues (also see A). N and C indicate the amino- and carboxyl-terminals, respectively.

The 3F scaffold used in this study is a novel snake α-neurotoxin, MicTx3, isolated from the South American coral snake, *Micrurus corallinus*. The cysteine framework of this protein is conserved and is similar to that of other short α-neurotoxins [10] (Figure 2A). Biochemical characterization of MicTx3 provided its affinity (29.5 ± 7.9 nM) for acetylcholine binding protein (AChBP), and electrophysiological studies of its inhibition characteristics were determined by the blockage of nicotinic acetylcholine receptor (nAChR) α7 currents expressed in *Xenopus *oocytes (data not shown). The amino acid residues forming the loops and β-sheets were deduced by secondary structure prediction, together with information from the literature regarding conserved amino acid residues in different toxins involved in binding to the nAChR [15,22]. Computer modeling of MicTx3 by superimposition of its spatial structure (β-sheets and loops) on those of other neurotoxins was found to be in good agreement (Figure 2B). The amino acids predicted to form the loops, including those putatively involved in binding to acetylcholine receptor, were subjected to randomization (Figure 2C). Residues T5-P10 in the Loop I (6 residues), K25-V34 in the loop II (10 residues) and A46-H52 in the loop III (7 residues) were randomized and a library constructed (Figure 2A and 2D). The amino acid residues predicted to form the β-sheets were left unaltered.

The full construct and sequences adopted for cDNA display of the 3F protein library are detailed in Additional file 1, Fig. S1 and Additional file 2, Table S1 online. After transcription, cell-free translation, cDNA display, purification and quantification, an estimated 1.2 × 10^11 ^molecules was obtained and used for the affinity selection of IL-6R (Figure 1A).

**Figure 3:**
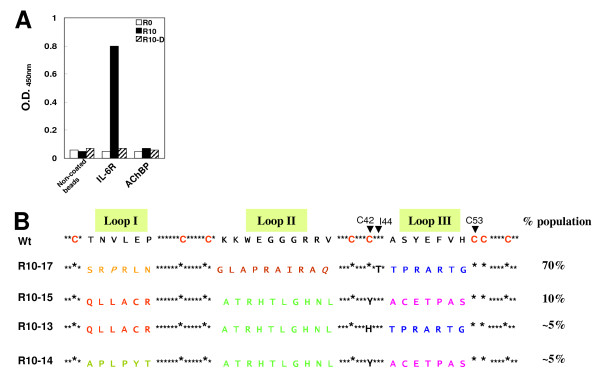
**Binding assay assessment of the quality of the libraries, and the sequences selected**. (A) The initial library (R0) and Round 10 library (R10) was prepared by the cDNA display method (up to step 4, Fig. 1A). The R10 library was reduced with dithiothreitol (DTT) to obtain the R10-D library. All three forms were incubated with non-coated beads, IL-6R coated beads or AChBP coated beads, washed and detected with Penta.His HRP (see Methods). (B) Sequences of the selected molecules compared to the native 3F sequence (shown as 'wt'). The loop sequences that were randomized are shown as letters and the non-randomized β-sheet residues are shown as asterisks. The cysteine residues are shown in red (C42 and C53 are indicated by arrows). '% Population' indicates the percentage of selected molecules out of the 30 clones that were sequenced. For example, approximately 70% of the R10-17-like proteins were selected. The sequences were assigned to respective groups (such as R10-17-like) based on the mutations observed in the loops (shown in italics). Similar loop sequences are shown in the same color. Mutations in the non-randomized portions are indicated by the mutated residues. C42 and I44 were found to be mutated to Y/H and T residues, respectively.

The binding characteristics of the 3F initial library (R0) were determined by the affinity of the library for immobilized AChBP (a binder of the parental MicTx3) and IL-6R. It was found that the library binds negligibly to both IL-6R and AChBP (Figure 3A) suggesting that the library does not bind to the receptors via the constant regions (*i.e.*, non-randomized β-sheet residues). These characteristics ensure that the library is suitable for generating novel binding molecules by randomization of the loops, followed by screening to identify the binding proteins.

### *In vitro *selection of the 3F library

The potential of the 3F library was assessed by searching the library for novel modulatory molecules of IL-6R. This was performed by affinity selection of binding molecules from the initial library over several rounds during which conditions were progressively made more stringent (see Methods). After 10 rounds of rigorous affinity selection, the DNA pools were cloned and sequenced.

The qualitative binding characteristics of Round 10 library (R10) were analyzed using IL-6R and AChBP (Figure 3A). The R10 displayed proteins showed negligible affinity for non-coated beads but bound significantly to IL-6R, indicating that the R10 library was enriched in comparison with R0, which did not bind to IL-6R. No detectable binding was observed to AChBP. Binding of the 3F-derived proteins is thus specific to IL-6R. Reduction of the R10 library with DTT resulted in no binding to any of the proteins tested, indicating that binding takes place via the protein part of the displayed protein (*i.e*., mRNA-protein) and the protein loop(s) protruded by forming disulfide bridges between cysteine residues may contribute to the specific binding.

Analysis of the sequences revealed that the pool could be majorly divided into 4 groups based on the similarity of the loop sequences (Figure 3B). R10-17-like sequences were 70% of the total clones, followed by R10-15, R10-13 and R10-14, at 5-10% each. A few mutations were observed in the constant regions, which could be due to failure of the polymerase proofreading mechanism as the library underwent totally ~650 cycles of PCR. Point mutations were also observed in the loop sequences, indicating that different amino acid residues may be accommodated in these regions. Silent mutations (different coding sequences expressing the same amino acid) were also observed at some positions. These observations support sequence specific enrichment of sequences from the library. C42, which is supposed to form a disulfide bond with C53 in the parent 3F, was found to be mutated to either Y or H in R10-13, 14 and 15. However, a cysteine residue (C47) was observed in the loop sequences of R10-14 and 15, which were subjected to randomization, so there is the possibility of a disulfide bond between C47 in the third loop and C53 in the β-sheet of the scaffold. In R10-13, no cysteine residue was selected either at the 47^th ^position or any of the other randomized positions in the third loop, which could have implications in the folding of the molecule. The cysteine framework was intact in R10-17 which was in majority.

### Expression of candidate proteins

**Figure 4:**
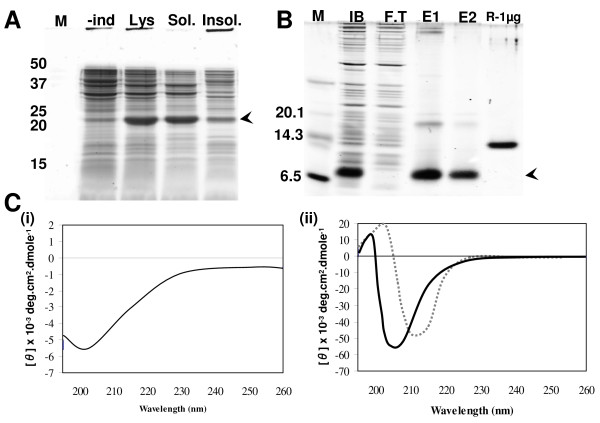
**Expression of candidate proteins in *Escherichia coli *and analysis by circular dichroism**. (A) Proteins were expressed as thioredoxin (Trx) fusions in *E. coli*. One of the candidates shown here, R10-14, was expressed 80% or more in the soluble fraction at an expression level of ~100 mg/l. The arrow indicates the R10-14 fusion protein. -Ind denotes uninduced culture, Lys denotes lysate that contains the total protein after cell lysis, Sol is the soluble fraction after cell lysis and separation of the pellet and Insol is the fraction which contained the solubilized pellet. (B) Expression of proteins without fusion. Candidates were cloned in pCOLDII vector and expressed in *E. coli *after cold shock (4°C) and growth at low temperature (15°C). The expression levels were in the range 10-40 mg/l, and most of the proteins were found in the insoluble fraction. The insoluble fraction was processed to obtain inclusion bodies and purified on a Ni-NTA affinity column. The expressed and purified protein is shown as IB, denoting inclusion body, F.T denotes flowthrough, and E1 and E2 are eluates 1 and 2, respectively. R-1 μg denotes the reference lysozyme used at 1 μg. (C) Circular dichroism spectra of the refolded candidate proteins. Candidate proteins R10-13 and 14 were refolded by two methods. In the first, the reduced proteins were refolded in the presence of reduced and oxidized glutathione by dialysis as shown in (i) and in the second, the proteins were refolded in the presence of the redox system (glutathione) and immobilized protein disulfide isomerase (ii). Both spectra are from R10-14.

Recombinant proteins were expressed in *Escherichia coli *by two approaches. In the first, the candidates were fused to the C-terminal of thioredoxin (Trx) and, in general, more than 50% of the expressed protein was soluble fraction. In the case of R10-14, more than 80% soluble protein was observed (Figure 4A). In the second, proteins were expressed without the fusion and were mostly observed in the insoluble fraction. The insoluble proteins were recovered as inclusion bodies, purified and refolded under various conditions (Figure 4B and 4C). We found that proteins refolded in the presence of reduced and oxidized glutathione (a redox pair) had random conformation, as judged from their CD spectra (Figure 4C (i)). However, when immobilized protein disulfide isomerase (PDI), a chaperone known to assist and improve folding/refolding [23], was included in the redox buffer, we observed spectra resembling β-sheets (Figure 4C (ii)). Ellipticities near 200 and 215 nm (spectra in dotted line) indicate a major β-sheet conformation [24]. The fusion and refolded proteins were used for biochemical assays and only the refolded proteins were used for biological assays.

### *In vitro *biochemical and biological assays

The dissociation constant (*K*_d_) was determined by the method described by Friguet *et al*. [25] with a few modifications. The values were found to be in the 48-115 nM range (Table 1), which are comparable to the dissociation constants of IL-6 (20 ± 3; Table 1).

**Table 1:** Characteristics of the selected 3F proteins and their derivative peptides.

No.	Name	**Sequence**^**1**^	**aa**^**2**^	Disulfide	***K***_**d **_**(nM)**^**3**^	**Function**^**4**^	**IC50**^**5 **^**(nM)**	**IC50/EC50**^**6 **^**(μM)**
1	R10-17	SRPRLN GLAPRAIRAQ TPRARTG	70	-	152 ± 10	NC	NC	-
								
2	R10-15	QLLACR ATRHTLGHNL ACETPAS	70	-	115 ± 9	C and NI	102 ± 13	NI
								
3	R10-13	QLLACR ATRHTLGHNL TPRARTG	70	-	48 ± 8	C and I	113 ± 7	1.4 ± 0.3
								
4	R10-14	APLPYT ATRHTLGHNL ACETPAS	70	-	55 ± 6	C and A	164 ± 9	0.02 ± 0.006
								
5	13-L1	LVCYQLLA**S**RPGTLET**G**PDDFTCV	24	C3 and C23	ND	I	ND	10 ± 1.5
								
6	13-L2	ETCPDDFTGVATRHTLGHNLTQYCS	25	C3 and C24	ND	NI	ND	NI
								
7	13-L3	ACAIPTPRARTGC	13	C2 and C13	ND	NI	ND	NI
								
8	14-L1	LVCYAPLPYTPGTLET**G**PDDFTCV	24	C3 and C23	ND	A	ND	0.03 ± 0.005
								
9	14-L3	IPACETPASC	10	C4 and C10	ND	NI	ND	NI

^1^Loop sequences of the proteins are for R10-17, 15, 13 and 14. Sequences of individual peptides are shown in full. 13-L1 represents loop 1 of the protein R10-13, bold letters indicate point mutations, and loop sequences are underlined.

^2^The number of residues in the proteins (*e.g*., 70) includes 61 residues of the protein, spacer GGS and 6xHis.

^3^Affinity was measured by Elisa (25). IL-6 was also assayed and the *K*_d _value was found to be 20 ± 5 nM. ND = not determined.

^4^NC = Non-Competitive, C = Competitive, NI = Non-Inhibitory, I = Inhibitory, A = Agonist.

^5^IC50 was determined by ELISA. IL-6 was also assayed and the value was found to be 80 ± 3 nM.

^6^Measured by IL-6 dependent DS-1 proliferation assay. IC50 = concentration for 50% growth inhibition, which for monoclonal antibody was determined to be 1.2 ± 0.15 nM and ED50 = 50% effective concentration, which for IL-6 was found to be 15 ± 2 pM.

The ability of the selected proteins to inhibit the interaction of IL-6 with IL-6R was analyzed by an *in vitro *biochemical competition assay. We observed that IL-6 inhibited the binding of biotinylated IL-6 to IL-6R with an IC50 value of 80 ± 3 nM, while the values obtained for R10-13, R10-14 and R10-15 were 113 ± 7, 164 ± 9 and 102 ± 13 nM, respectively (Table 1). R10-17 was found to be non-competitive.

**Figure 5:**
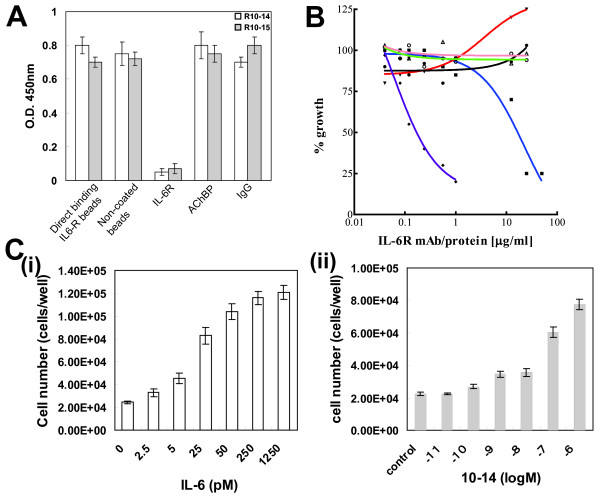
**Characteristics of the selected candidate proteins**. (A) Specificity analysis by competition assay. The candidates (R10-14 and 15 shown here) at 100 nM were incubated with 1 μM IL-6R, AChBP or IgG. The unbound proteins were captured by immobilized IL-6R and detected using Penta.His HRP. Non-coated beads were used as negative controls and IL-6R beads were used as positive controls (direct binding). A low signal indicates the specificity of the candidates for IL-6R, while a high signal indicates no apparent affinity for AChBP or IgG. (B) Assessment of the candidates by biological assay. The candidate proteins were assessed for their potential to inhibit and/or enhance the proliferation of DS-1 cells (a human B-lymphoma cell line). IL-6R monoclonal antibody was used as a positive control for the inhibition of proliferation. Graphs are plotted as concentration of IL-6R mAb and peptides vs. % Growth. Open circles denote control (PBS in which IL-6R mAb and proteins were dissolved), filled diamonds denote IL-6R mAb (purple line), filled squares denote R10-13, inverted filled triangles denote R10-14, filled circles denote R10-15 (black line) and open triangles denote R10-17. (C) Assessment of the agonist activity of R10-14. The candidate protein, R10-14, which enhanced the proliferation of cells in (B), was assessed for its potential to enhance cell proliferation in the absence of IL-6; (i) shows the dose-dependent proliferation of cells by IL-6 and (ii) shows the dose-dependent proliferation of cells by R10-14. Proper controls such as PBS were included. ED50 for IL-6 and R10-14 were determined to be 15 ± 2 pM and 20 ± 6 nM, respectively.

The selected proteins, R10-14 and R10-15, were found to be highly specific to IL-6R, with no apparent affinity for other proteins such as AChBP and IgG (Figure 5A).

Next, we carried out *in vitro *biological assays using the IL-6-dependent DS-1 human B-lymphoma cell line. As shown in Figure 5B, IL-6R mAb inhibited the IL-6 dependent proliferation of cells in a dose dependent manner (IC50 = 1.2 ± 0.15 nM), and R10-13 was found to inhibit the growth of cells (IC50 = 1.4 ± 0.3 μM). The other proteins, R10-15 and 17, showed essentially no effect on the cell growth. However, we observed that R10-14 boosted the proliferation of cells and thus speculated that this molecule could be an agonist of IL-6R. Therefore, we further investigated the potential of R10-14 to induce cell proliferation in the absence of IL-6 and found that R10-14 indeed induces proliferation even in the absence of IL-6 and thus acts as an IL-6 mimetic molecule (Figure 5C, (i) and 5C (ii)). The ED50 (50% effective dose) values were determined to be 15 ± 2 pM for IL-6 and 20 ± 6 nM for R10-14 (Figure 5C and Table 1).

### Size optimization of the three-finger scaffold (3F to 1F)

**Figure 6:**
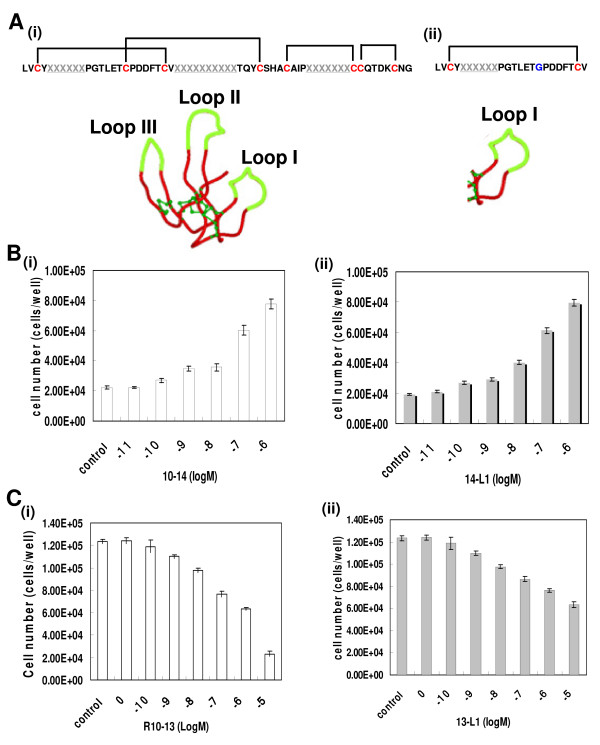
**Biological assessment of the size-optimized peptides from the selected candidate proteins**. (A) The size optimization strategy was designed to obtain peptides resembling three single fingers between 12-25 residues long (see Additional file 3 Fig. S2) from the parent candidate proteins which are 61 residues long (i). The spatial structures of the parent protein (i) and the derived peptide containing Loop I (ii) are given (disulfide bonds are shown in green and the fluorescent green denotes different loop sequences. (B) Assessment of the peptides as agonists of IL-6R. R10-14 (i) and the designed and chemically synthesized peptides 14-L1 (ii) were assessed for their ability to stimulate the proliferation of DS-1 cells in the absence of IL-6 in a dose-dependent manner. (C) Assessment of the peptides as antagonists. R10-13 (i) and the designed and chemically synthesized peptides 13-L1 (ii) were challenged in the IL-6 dependent proliferation of DS-1 cells in a dose-dependent manner.

Short peptides retaining the essential features of the 3F scaffold were generated by size minimization. Peptides were designed that included the sequences of the scaffold forming the β-sheets, loops and the disulfide bonds of the selected molecules (see Methods). Thus, the resulting peptides resemble one of the fingers (1F) of the three-finger protein (3F) (Figure 6A and Additional file 3, Fig. S2 online).

The potency of the designed peptides was assessed using the biological assay described above. In the case of R10-14 derived peptides, 13-L2 (which is identical to the 2^nd ^finger of R10-14; also see Figure 3B) and 14-L3 were ineffective in inducing cell proliferation. However, 14-L1 (ED50 = 30 ± 5 nM) was as effective as the parent molecule, R10-14 (ED50 = 20 ± 6 nM), at inducing cell proliferation. Similarly, in the case of R10-13 derived peptides, 13-L2 and 13-L3 could not inhibit cell proliferation, however 13-L1 inhibited cell proliferation (IC50 = 10 ± 1.5 μM), albeit with a lower potency than the parent R10-13 (IC50 = 1.4 ± 0.3 μM) (Figure 6B, 6C and Table 1).

## Discussion

Antibodies and its derivatives such as scFv, minibody, diabody and others have been increasingly used for protein engineering and directed evolution due to their high affinity and specificity towards their targets [26]. Recently, however, alternate protein scaffolds that provide potential advantages over antibodies such as smaller size, easier production and optimization, are showing promise and are attracting attention [8,9]. Such scaffolds are comprised of a single polypeptide chain, have defined structure, and contain amenable regions such as loops that resemble the complementary determining regions (CDR) of antibodies. These CDR-like loops can be utilized to generate large libraries that can be used to obtain molecules with completely novel properties.

In this regard, we identified and explored a new scaffold, three-finger (3F), for directed evolution and protein engineering. 3F proteins are small, compact, highly specific, stable to thermal and protease exposure, and are found in a variety of organisms ranging from snakes to humans [10-13]. Furthermore, molecular evolution study revealed that the 3F toxin genes show accelerated evolution, by which the gene product could rapidly adapt to the environmental changes including prey species. The evolution rates are significantly higher in the finger regions. These characteristics make them excellent candidates for protein engineering and directed evolution and, thus, potentially useful for research, diagnostic and therapeutic uses. We have utilized cDNA display for the directed evolution of a 3F scaffold library to obtain molecules that bind to and possibly regulate interleukin-6 receptor (IL-6R).

cDNA display is based on the formation of a covalent fusion between the expressed protein and puromycin linked to the genotype (cDNA). Briefly, a DNA linker containing puromycin is rapidly ligated to the mRNA. Following protein expression, puromycin forms a covalent fusion with the protein on the stalled ribosome [4,5]. This is followed by rapid purification of the complex, facilitated by the biotin-streptavidin pair, and subsequent cDNA synthesis utilizing the 'built-in' primer contained in the linker. Thus, the protein, which is covalently attached to the puromycin-linker, eventually gets linked to the cDNA (Figure 1A) [6]. This process is extremely fast, prevents degradation of the mRNA portion and thus contributes to prepare the library intact in quality and size, which are crucial for the subsequent selection of molecules. The cDNA display technology utilizes a cell-free translation system and, as a result, is capable of generating libraries in the order of 10^12-14^, compared to 10^8 ^in the case of cell-dependent methods such as phage display [3].

Affinity selection of the 3F library aimed at identifying novel molecules binding to IL-6R enriched the library with proteins specific for IL-6R; the sequences of these proteins were divided into different major and minor groups (Figure 3). The selected candidates bound to IL-6R with affinity (nanomolar range) and specificity similar to that of antibodies in the biochemical assays. Candidates that competed with the natural ligand, IL-6, were also successfully obtained. Antagonists as well as agonists were identified biologically using an IL-6 dependent cell proliferation assay (Table 1). These results demonstrate the potentials of the 3F scaffold to accommodate residues, different from the parent residues, which are required to generate high-diversity libraries and then to utilize the libraries for obtaining novel ligands for macromolecular targets such as IL-6R by iterative selection. Disulfide-rich proteins such as knottin, tendamistat and others have been shown to be useful as templates to search for protease inhibitiors, however, none have been shown to bind and/or regulate the activity of macromolecular receptors [8]. Our results show for the first time that regulatory molecules against receptors can be obtained by the directed evolution of the 3F scaffold.

The 3F scaffold provides potential advantages over related scaffold proteins that have been successfully used for directed evolution. 3F (~6.7 kDa) is smaller than many other proteins such as scFv (~30 kDa), DARPin (~20 kDa), lipocalin (~20 kDa), fibronectin (~10 kDa) and zinc-finger (~9 kDa), and is comparable to Affibody (~6 kDa); these proteins have been used for the purpose of directed evolution [9]. The number of residues in the loops that can be utilized for the construction of libraries is around 38%, which is larger than that of published scaffolds such as ~10% (scFv) [26], ~22% (Affibody) [27], ~22% (fibronectin) [28], ~26% (zinc-finger) [29], ~9% (lipocalin) [30], ~21% (DARPin) [31], and others. The greater flexibility regarding the residues that can be randomized provides a larger interaction surface between the binding partners during the binding event. The discovery of an agonist in the 3F library also demonstrates the utility of this approach, particularly from the viewpoint that agonists have been identified from immunoglobulin sources [32-34] and random peptide libraries [35,36], but there are no published reports of agonistic molecules from non-immunoglobulin proteins [9]. The high affinity and remarkable specificity of 3F proteins for their targets, along with their stability, make this scaffold suitable for diagnostic, therapeutic and protein chip purposes. Furthermore, the presence of a single lysine (K58) near the C-terminus in the 3F molecule used in this study will facilitate homogeneous labeling of the 3F proteins with chemical moieties such as fluorescein, biotin and other labels and probes that can be exploited for research and diagnostic purposes (*e.g*., fluorescence imaging of tumors, protein chips, ELISA).

When the candidate proteins were expressed in *E. coli *as recombinant proteins in fusion with thioredoxin, more than 50% of the expressed proteins were found in the soluble fraction; one of the candidates, R10-14, showed more than 80% soluble protein (Figure 4A). Similarly, the candidates also expressed well at 1-2 mg/ml of cell-free lysate when produced as maltose binding protein fusions in a bacterial cell-free translation system such as RTS (Roche, Penzberg, Germany), with nearly all of the protein present in the soluble fraction and checked for their binding activities for IL-6R (data not shown). In contrast, disulfide-rich proteins are known to be poorly expressed in the native form in bacteria, and generally tend to form inclusion bodies [37]. In an attempt to produce non-fusion proteins in bacteria, we observed that the proteins aggregated as inclusion bodies (Figure 4B). However, by using a refolding system that included the redox buffer and immobilized protein disulfide isomerase, the proteins seem to be refolded from the analysis of circular dichroism (Figure 4C). Further, 3F proteins can also be successfully produced in the native state using *Pichia pastoris *as the host system [38].

The 3F proteins are leaf-like, flat molecules with the three fingers extending from a globular head [39,40]. Taking advantage of this structure, we designed a strategy to minimize the size of selected 3F proteins into three, single finger peptides, which lead to three interesting results. First, the size was successfully reduced to 24 residues compared to 61 residues in the parent 3F, and thus can be conveniently prepared by chemically synthesis and facilitate molecular dynamic studies; second, we identified the effector fingers which retained activity; third, function was observed in the 1^st ^finger, *i.e.*, 13-L1 and 14-L1 (Table 1).

Scaffolds based on single-chain repeat proteins such as DARPins and avimers have been successfully moderated in size (reduced or increased) due to the modular nature of parent proteins [31,41], however, there are no reports of other single-chain proteins, such as fibronectin, lipocalins and affibodies, being successfully reduced in size. The possibility of further size reduction of monomers in DARPins (the monomer consists of 33 residues) and avimers (a monomer containing 35 residues) has also not been investigated. Therefore, we believe that this is the first report of the successful reduction of a single-chain 3F scaffold to shorter peptides of only 24 residues which retain activity comparable to that of the parent protein. Recently, ~3 kDa peptides derived by rational design from V_H _(complementarity region) and V_L _(framework region) domains of Fab have been shown to mimic the parent antibody [42]. Encouraged by these results, the concept of single finger libraries will be interesting to pursue and might be more attractive.

IL-6 is a pleiotropic cytokine involved in the regulation of cell growth, differentiation and various other cellular functions [17], and is known to play a role in the pathogenesis of a variety of diseases [18,20,43-46] and in brain injury [47,48]. Therefore, both antagonist and agonist molecules that can regulate the IL-6R-mediated signaling of IL-6 are important for research, diagnostic and therapeutic purposes. An antibody that acts as an antagonist of IL-6R has been reported [49]; however, there are no reports of agonists of IL-6R. The present study generated R10-13, which has a size 1/20^th ^that of an antibody and an IC50 of 9.8 μg/ml (IC50 = 0.18 μg/ml of mAb, as determined in this study), and also 13-L1, which has a size 1/57^th ^that of an antibody, and an IC50 = 26 μg/ml. In different reports, IC50 values for peptides selected by phage display and rational design were reported in the range 25-60 μg/ml [50,51]. 3F derived peptides are, therefore, potentially capable of inhibiting the IL-6R-mediated signaling. R10-14 (ED50 = 140 ng/ml) and 14-L1 (ED50 = 79 ng/ml) can potentially be developed and used as agonists of IL-6R (ED50 = 0.5 ng/ml of IL-6 as determined by this assay) either to supplement IL-6, or used when IL-6 is deficient.

It is noteworthy that the directed evolution of 3F library which was carried out using the soluble extracellular IL-6R generated molecules having comparable affinity and competitive potential to IL-6 (Table 1). However, in cellular assays the peptides were found to be less potent either as antagonist or agonist compared to IL-6R mAb and IL-6 showing approximately 100 times lesser activity. The antagonist and agonist molecules developed in this study, however, have comparable or better efficacy than those previously reported. Martin *et al*. obtained a clone from affibody library with an affinity in the micromolar range and antagonist function [52], Su *et al*. [50] and Feng *et al*. [51] reported peptides with IC50 in the range 25-60 μg/ml. Further, phage display based selections of constrained cyclic libraries have been reported to generate agonists in the micromolar range [35,36]. The above peptides and proteins obtained in this study may be regarded as lead molecules from the viewpoint of potency, which can be increased further by exploiting affinity maturation of the parent 3F candidates [53,54] optimization by dimerization or multimerization of the peptides [55-57], and other approaches. These lead molecules were generated by directed evolution of a 3F protein library. Therefore, 3F library used in this study provides a platform to obtain regulatory molecules of IL-6 receptor and, in principle, other macromolecules.

In analogy to 3F scaffold, other peptide scaffolds originating from accelerated evolution such as inhibitor cysteine knot may also be utilized as templates for random peptide libraries.

## Conclusions

We have constructed a high diversity library utilizing 3F scaffold as a template and randomizing the loops with which the parent molecule binds to its natural receptor. This library was screened against soluble extracellular IL-6R and yielded ligand molecules that bind with high affinity to the receptor comparable to IL-6. These ligands have both antagonist and agonist function as revealed in a cellular assay. We have also successfully achieved size reduction of this single chain scaffold into single fingers without significant loss of activity. Therefore, 3F proteins are promising candidates for protein engineering, and directed evolution can be used to generate molecules for research, diagnostic and therapeutic uses.

## Methods

### Preparation of the three-finger library

A full construct for preparing the three-finger (3F) protein library was designed to facilitate the formation and purification of cDNA displayed proteins. The 3F genetic construct was prepared by joining the fragments by overlap PCR (see Additional file 2, Table S1 and Additional file 1, Fig. S1 online). Briefly, to the sequence of the 3F library, SP6 fragment containing the SP6 promoter, cap site, *Xenopus *globin untranslated sequence (UTR) and translation initiation site was added at the 5' end, while the (G_3_-S)_2 _spacer, C-terminal 6xHis, (G_3_-S) spacer and Y-tag sequences were added at the 3' end (Additional file 1, Fig. S1). The final library was expressed and purified by Ni-NTA agarose (Qiagen, Valencia, CA, USA) and the library's quality was assessed by sequencing 20 clones picked at random after cloning them into the TA cloning vector (Invitrogen, Carlsbad, CA, USA).

### Transcription of library DNAs

The above library DNAs were subjected to polyacrylamide gel electrophoresis (PAGE), purified from the gel, and then annealed by heating at 94°C and gradient cooling to form the correctly paired double-stranded DNA. The template DNAs were transcribed by SP6 RNA polymerase in a RiboMAX Large Scale Production Systems (Promega, Madison, WI, USA). Reactions were terminated by adding DNase I and products were purified using the phenol/chloroform method. RNA concentration was measured by a spectrophotometer at 260 nm.

### Synthesis of the puromycin-linker

The puromycin-linker was synthesized, as described previously [6], by cross-linking two modified oligonucleotides with the hetero-bifunctional reagent, EMCS (N-(6-maleimidocaproyloxy) succinimide) (Figure 1B).

The modified oligonucleotides, Puro-F-S [5'-(S)-TC (F)-(Spec18)-(Spec18)-(Spec18)-

(Spec18)-CC-(Puro)-3'] and Biotin-loop [5'-CCCGGTGCAGCTGTTTCATC(T-B) CGGAAACAGCTGCACCCCCCGCCGCCCCCCG(T)CCT-3'] were custom synthesized (BEX Co., Tokyo, Japan). The symbol (S) denotes 5'-thiol-modifier C6, (F), fluorescein-dT; (Puro), puromycin CPG; (Spec18), spacer phosphoramidite 18; (T), amino-modifier C6 dT; (T-B), biotin-dT. The underlined sequence indicates the cutting site of restriction enzyme *Pvu*II) All phosphoramidite reagents used for modifications were from Glen Research (Sterling, VA, USA).

### Ligation of mRNA to the linker

mRNA was annealed to the biotinylated puromycin-linker DNA (1:1 ratio) via the Y-tag sequence in 1× ligase buffer (Takara, Kyoto, Japan) by heating at 94°C and cooling slowly. mRNA and linker were ligated by the addition of T4 kinase (3 U) and T4 RNA ligase (20 U) (Takara) at 25°C for 1 h, then the conjugated product was purified using an RNeasy Kit (Qiagen). Ligation efficiency and the purity of the products were checked by polyacrylamide gel electrophoresis using FITC and/or VistraGreen (Molecular Probes, USA) staining on a fluoroimager (Bio-Rad, Hercules, CA, USA).

### cDNA display

mRNA-puromycin linker DNA (3-5 picomoles) was translated in 25 μl using a Rectic Lysate IVT Kit (Ambion, Austin, TX, USA) at 30°C for 10 min. After translation, proteins were covalently linked to the puromycin linker in the presence of 65 mM MgCl_2 _and 750 mM KCl at 37°C for 2 h. Purification was achieved using the biotin-streptavidin interaction for 10-15 min., followed by M-MLV reverse transcriptase (Takara) reaction to form the cDNA/mRNA hybrid utilizing the 'built-in' primer included in the linker (Figure 1B). Displayed proteins were released from the biotin-streptavidin complex upon digestion with 12 U of *Pvu*II restriction enzyme in the supplied buffer at 37°C for 1 h. Full length displayed proteins were purified utilizing the C-terminal 6xHis-tag by Ni-NTA magnetic beads (Qiagen). The purified display proteins were quantified by fluorescence using a Beacon 2000 (Panvera, WI, USA) and a known FITC-labeled DNA as standard.

The whole process was scaled up for library preparation where 200 pmols was used for translation; the subsequent rounds were reduced to 1/5^th ^for R2-R5 and 1/10^th ^for R6-R10.

### Library quality assessment

The affinity of the initial 3F library (R0) was checked by a binding assay for immobilized IL-6R and AChBP (see immobilization procedures in the next section). Equal amounts of 3F library prepared by cDNA display (up to step 4, Figure 1A) were incubated with non-coated beads, 250 nM IL-6R or AChBP coated beads for 1 h at room temperature in PBS. Each mixture was washed with PBS-T (0.1% Tween) and subsequently incubated with Penta.His-HRP (Qiagen) in PBS-T for 30 min. at 25°C. The mixtures were washed several times with PBS-T followed by the addition of substrate, 3, 3', 5, 5'-Tetramethylbenzidine (TMB; Sigma, Saint Louis, USA). After color development was complete, the reaction was stopped by the addition of 0.5 M H_2_SO_4_, each mixture was centrifuged, and absorbance was measured at 450 nm.

### Preparation of immobilized proteins

Extracellular IL-6R (PeproTech, London, UK), AChBP (cDNA was cloned from *Aplysia kurodai *[manuscript in preparation] and the recombinant protein was expressed in *E. coli *and purified in our laboratory), IgG (human immunoglobulin; Sigma) and BSA (Ambion) were immobilized on NHS-activated Sepharose 4 Fast Flow (Amersham Biosciences) utilizing the amine coupling chemistries to form a chemically stable amide bond according to manufacturer's instructions. Coupling efficiency was estimated by absorbance measurements of initial and unbound protein at 280 nm. The activity of the immobilized proteins (except for BSA) was measured by ELISA using either biotinylated or 6xHis containing ligands. Non-coated beads were also prepared by the same derivatization procedure, but in the absence of protein.

### Selection of IL-6R binders

The above 3F library was screened to identify molecules selectively binding to IL-6R. Each round of selection was performed with the non-coated beads and IL-6R coated beads in a batch-selection mode using Phosphate Buffered Saline (PBS) containing an additional 100 mM NaCl and 0.1% Tween-20. The initial round contained 2 nM cDNA displayed protein and 300 nM IL-6R. Prior to the selection of IL-6R binders, non-specific Sepharose binders were excluded by preincubation of the library with non-coated beads for 1 h. This was followed by incubation of this precleared library with IL-6R coated beads for 1 h. After incubation, the IL-6R beads were washed several times with the PBS/NaCl/Tween-20 buffer and eluted with the same buffer containing 100 mM DTT (dithiothreitol, Sigma). The eluted products were desalted and PCR amplified (conditions described above) with PCR forward and reverse primers (see Additional file 2, Table S1 online), purified and used as templates for PCR amplification with the SP6 fragment. The DNAs were gel purified, transcribed, mRNA ligated to puromycin linker, translated, transformed into display proteins, reverse transcribed, Ni-NTA affinity purified, quantified and used for the next round of selection. The concentration of IL-6R and the incubation time were gradually reduced from 300 nM to 30 nM and from 1 h to 15 min, respectively, while the number of washes increased progressively during further rounds of selection in order to apply selection pressure. In round 9 and 10, 30 nM biotinylated IL-6R was used.

After 10 rounds of stringent selection the eluted pool was PCR amplified, cloned into TA cloning vector (Invitrogen) and sequenced.

### Assessment of the selection

Direct binding analysis using displayed proteins: Selected Round 10 library (R10) was prepared by the cDNA display method (up to step 4 of Figure 1A) and purified by spin columns (Bio-Rad). One of the fractions was denatured by treatment with 100 mM dithiothreitol (DTT) for 1 h and purified (designated as R10-D).

Purified display proteins (R10 and R10-D; 5 μl) were mixed with non-coated beads and 250 nM of IL-6R or AChBP coated beads in PBS-BSA (0.01% BSA) and incubated at 25°C for 1 h. The mixtures were washed with PBS-T (0.1% Tween) and subsequently incubated with Penta.His-HRP (Qiagen) in PBS-T for 30 min. at 25°C. The mixtures were then washed several times with PBS-T followed by addition of the substrate, 3, 3', 5, 5'-tetramethylbenzidine (TMB; Sigma). After color development was complete, the reaction was stopped by the addition of H_2_SO_4_, the sample was centrifuged, and absorbance measured at 450 nm.

### Protein preparation

Proteins were prepared as thioredoxin fusions using the pBAD/TOPO Thiofusion expression Kit (Invitrogen). Thioredoxin (Trx) was also prepared for control experiments. The selected candidates were PCR amplified (encoding C-terminal 6xHis), cloned into pBAD/TOPO Thiofusion expression vector and transformed according to the manufacturer's instructions. Frames were analyzed by sequencing.

The positive clones were cultured in Luria-Bertani (LB) medium containing 50 μg/ml ampicillin at 37°C for 16 h. A small portion of the culture was transferred to fresh LB-ampicillin medium and grown to an O.D. of 0.5 at 600 nm, then the culture was induced with 0.02% arabinose for 4 h at 37°C. The cells were harvested by centrifugation at 3000 rpm for 20 min and lysed with Bugbuster Protein Extraction Reagent (Novagen, San Diego, USA) in the presence of 1 μg/ml DNase, 1 μg/ml RNase and protease inhibitor cocktail (Sigma) at 25°C for 20-30 min. The lysate was centrifuged and separated into supernatant (soluble fraction) and pellet (insoluble fraction) and analyzed on SDS-PAGE. The soluble fraction was purified by Ni-NTA (Qiagen) affinity for the 6xHis tag of the expressed peptide under native conditions to near homogeneity. Proteins were also confirmed by Western blotting for 6xHis by Penta.His HRP (Qiagen). Protein concentration was measured using a Bio-Rad Protein Assay Kit (Bio-Rad) with bovine serum albumin (BSA) as a standard, and by densitometry measurements of the protein bands on SDS-PAGE gels using known protein concentration standards such as Trx.

Non-fusion proteins were prepared by cloning the candidates in pCOLDII vector (Takara). The plasmids were transformed into Rosetta-gami (DE3) pLacI (Novagen). For expression, colonies were grown in LB medium containing 50 μg/ml ampicillin at 37°C for 16 h. A small portion of the culture was added to fresh LB-ampicillin medium and grown to an O.D. of 0.5 at 600 nm. The culture was incubated at 4°C for 30 min. and induced with 0.1 mM IPTG for 15 h at 15°C. The cells were harvested by centrifugation at 3000 rpm for 20 min and lysed with Bugbuster Protein Extraction Reagent in the presence of 1 μg/ml DNase, 1 μg/ml RNase and protease inhibitor cocktail at 25°C for 20-30 min. The lysate was centrifuged and separated into soluble and insoluble fractions. The insoluble fraction was purified to provide inclusion bodies, dissolved in buffer containing 50 mM CAPS, pH 11.0, 0.3% N-lauroylsarcosine and 0.1% β-mercaptoethanol (Sigma), purified on a Ni-NTA affinity column under denaturing conditions to near homogeneity, analyzed on tricine-PAGE and confirmed by Western blotting for 6xHis. Lysozyme was used as a concentration reference.

The denatured proteins were refolded in the presence of 1 mM oxidized glutathione and 10 mM reduced glutathione (Sigma). The proteins were also refolded under identical redox conditions in the presence of immobilized protein disulfide isomerase (PDI; Takara) at a ratio equimolar to the soluble protein. Protein concentration was measured with a Bio-Rad Protein Assay Kit (Bio-Rad) using bovine serum albumin (BSA) as a standard and/or by densitometry of protein bands on tricine-PAGE gels with known protein concentration standards such as lysozyme.

Proteins refolded under both conditions were analyzed by circular dichroism spectroscopy on a Jasco J-805 spectropolarimeter. Proteins were prepared in PBS at 0.3-0.5 mg/ml and measurements taken at 25°C from 195-260 nm.

### Binding and inhibition assays

1. Binding assay for dissociation constant (*K*_d_)

Binding affinity of the proteins was assayed as described by Friguet *et al*. [25] with several modifications. A constant amount of protein (10-25 nM) was incubated with varying amounts of IL-6R (1 nM-1 μM) in PBS-BSA (0.01% BSA) at 25°C for 1 h. Proper controls were included. The mixture was applied to a constant amount of IL-6R coated beads (200 nM) and incubated further for 30 min. After several washings with PBS-T (Tween-20, 0.1%), streptavidin horseradish peroxidase (SA-HRP; Amersham Biosciences) at 1/2000 dilution or Penta.His-HRP (Qiagen) at 1/1000 dilution was added and incubated for 30 min - 1 h. The supernatant was removed and the beads were washed 4-5 times with PBS-T. TMB substrate (200 μl) was added for color development and the reaction was stopped with 0.5 M H_2_SO_4_. Absorbance was monitored at 450 nm. Data were plotted using GraphPad Prism 4 (GraphPad Software Inc., San Diego, CA, USA).

2. Inhibition assay for IC50

Inhibition of the IL-6/IL-6R interaction by the proteins was assayed by competitive inhibition experiments. A fixed amount of biotinylated IL-6 (45 nM, *i.e.*, ~3 times the *K*_d_) and varying amounts of protein/peptide (1 nM - 2 μM) were incubated with a constant amount of IL-6R coated beads (350 nM) in PBS-BSA at 25°C for 1 h. After several washings with PBS-T, SA-HRP at 1/2000 dilution was added and incubated for 30 min. The supernatant was removed and the beads were washed 4-5 times with PBS-T. TMB substrate (200 μl) was added for color development, the reaction was stopped with 0.5 M H_2_SO_4 _and absorbance was monitored at 450 nm. Data were plotted using GraphPad Prism 4 (GraphPad Software Inc.).

3. Specificity assay

100 nM of proteins was incubated with 1 μM of various soluble proteins (IL-6R, AChBP or IgG) at RT for 1 h. The mixtures were further incubated with 300 nM of IL-6R coated beads for 1 h. The subsequent procedures were as described for the binding assay.

### Design of minimized peptides

Short cyclic peptides were designed based on the structure and disulfide pattern of 3F proteins. The sequences of the peptides are shown in Table-1. The highly conserved disulfide bonds in 3F proteins are C1-C3, C2-C4, C5-C6 and C7-C8 (numbered according to the position of the cysteine residues going from the N to the C terminus) [10]. Therefore, we designed cyclic peptides containing the individual loops sequences between C1-C3 (24 residues) where C2 was changed to G, C2-C4 (25 residues) where C3 was changed to G and C5-C6 (13 and 10 residues), respectively. If a cysteine was in the loop sequence selected from the library, it was replaced by serine (13-L1). In the case of 14-L3, due to mutation of the C5 residue, a disulfide bond was formed between the cysteine residue in the loop and C6. All the peptides were synthesized and purified by (Invitrogen, Tokyo, Japan).

### Cellular assay

Cell culture and reagents: The lymphoblast cell line DS-1 was obtained from the American Type Culture Collection (CRL-11102; Manassas, USA), and were cultured in RPMI 1640 complete culture medium (Gibco, New York, USA) supplemented with 10% fetal bovine serum (FBS; Gibco), 10 mM HEPES buffer (Gibco), 10 U/ml human IL-6 (Sigma, USA), penicillin (16 mg/l) and streptomycin (25 mg/l) at 37°C in a humidified incubator with 5% CO_2 _[58]. The cells were routinely passaged every 2 or 3 days in 35 mm flask (Falcon, Akasaka, Japan). Cells were continuously cultured until harvest for analysis.

Analysis of cell proliferation: Logarithmic growing DS-1 cells were dispersed with pipetting, centrifuged at 3000 rpm for 5 min, washed two times in phosphate buffer saline, and were finally resuspended in standard culture medium without IL-6. Prior to the assay, the DS-1 cells (~2 × 10^4 ^cells/ml) were cultured for 24 h in triplicate in 24 well, flat-bottomed microtiter plates (Falcon) in 1 ml of culture medium. The candidate peptides, IL-6 and anti-human IL-6R antibody (R & D Systems, Minneapolis, USA) were prepared in standard medium at desired concentration. After 24 h pre-cultivation, cells were treated with IL-6 (10 U/ml) and specific antibody (0.03 - 2 μg/ml), protein or peptide (10^-10 ^- 10^-5 ^M) for 48 h, and then the cells were counted under an inverted microscope (Olympus, Hatagaya, Japan).

## Competing interests

The authors declare that they have no competing interests.

## Authors' contributions

MN designed and performed most of the experiments, analyzed the data and wrote the manuscript. SK prepared the proteins, multiple alignment and protein structures. CT and TS performed cellular assays and wrote a part of the manuscript. MM and NN analyzed the data. TK conceived the study, analyzed the data and wrote the manuscript. All authors approved the final manuscript.

## Acknowledgements

The authors are grateful to Mr. Toshikatsu Kobayashi for his kind advices and generous cooperation. We thank Drs. T. Toda and Y. Tamura for cooperation; and J. Yamaguchi, S. Ohtaki and S. Honda for support. This work was supported by the Strategic Research Center (Super COE) Development Program of Special Coordination Funds for Promoting Science and Technology, Ministry of Education, Culture, Sports, Science and Technology (MEXT) and Innovation Center for Start-Ups, National Institute of Advanced Industrial Science and Technology, Japan and in part by New Energy and Industrial Technology Development Organization (NEDO), Japan.

## Supplementary Material

Additional file 1 **Construction of the three-finger library**. (A) Primary structure of the three-finger protein, MicTx3, used in this study. The four disulfide bonds formed between C1-C3, C2-C4, C5-C6 and C7-C8 are conserved among the three-finger proteins across a wide variety of species. The loops are shown in color and were randomized to generate the libraries. (B) Genetic construct adopted for cDNA display. SP6 contains the sp6 promoter, capping site and *Xenopus *globin untranslated sequence (UTR). ATG is the translation initiation codon. The 3F gene with the three randomized loops provides the basis of the library. G3S is spacer, 6XHis facilitates affinity purification by Ni-NTA, and Y-tag facilitates ligation of mRNA to the puromycin linker which contains the complementary Y-tag sequence. (C) Construction of library by overlap PCR. (i) The library was constructed by joining the fragments listed in supplementary Table-1 using overlap PCR in various steps. The portions marked in orange denote the randomized residues. (ii) Analysis of the PCR products by electrophoresis. The bands denote the respective fragments. (D) Direct sequencing of the PCR products of the library. The portions shown by arrows represent loops that were randomized. The peaks are smaller than normal due to the presence of a mixture of residues. Click here for file

Additional file 2 **List of oligonucleotides used for library preparation and amplification**. Oligonucleotides that were used in the preparation of 3F library and the primers required for amplification of the selected library are listed. Click here for file

Additional file 3 **Design of shorter peptides containing disulfide bonds from the parent 3F**. (A) Scheme for designing peptides. 3F is a leaf-like flat molecule with the three fingers extending from the globular head. The three fingers were designed to split into three individual fingers. (B) Design of the peptides. Primary sequence of the three-finger protein. The four disulfide bonds are formed between C1-C3, C2-C4, C5-C6 and C7-C8. The loops are located between the disulfide bonds. (ii) Short peptides from the 3F sequence. The sequences of each of the peptides correspond to one of the loops. In Loop-1 (L-1), C2 was replaced by G, and in L-2, C3 was changed to G to restore the disulfide bonds as in the parent 3F. In the case of R10-14, C5 was found to be mutated to Y, however, a cysteine residue was selected in the randomized loop. Therefore, a disulfide bond was formed between this cysteine and C6. Click here for file

## References

[B1] Pluckthun A Schaffitzel C Hanes J Jermutus L In vitro selection and evolution of proteins Adv Protein Chem 2000 55 367 403 full_text 11050939 10.1016/s0065-3233(01)55009-3

[B2] Smith GP Filamentous fusion phage: novel expression vectors that display cloned antigens on the virion surface Science 1985 228 1315 1317 10.1126/science.4001944 4001944

[B3] Lipovsek D Pluckthun A In-vitro protein evolution by ribosome display and mRNA display J Immunol Methods 2004 290 51 67 10.1016/j.jim.2004.04.008 15261571

[B4] Nemoto N Miyamoto-Sato E Husimi Y Yanagawa H In vitro virus: bonding of mRNA bearing puromycin at the 3'-terminal end to the C-terminal end of its encoded protein on the ribosome in vitro FEBS Lett 1997 414 405 408 10.1016/S0014-5793(97)01026-0 9315729

[B5] Roberts RW Szostak JW RNA-peptide fusions for the in vitro selection of peptides and proteins Proc Natl Acad Sci USA 1997 94 12297 12302 10.1073/pnas.94.23.12297 9356443 PMC24913

[B6] Yamaguchi J Naimuddin M Biyani M Sasaki T Machida M Kubo T Funatsu T Husimi Y Nemoto N cDNA display: a novel screening method for functional disulfide-rich peptides by solid-phase synthesis and stabilization of mRNA-protein fusions Nucleic Acids Res 2009 37 16 e108 10.1093/nar/gkp514 19528071 PMC2760808

[B7] Binz HK Pluckthun A Engineered proteins as specific binding reagents Curr Opin Biotechnol 2005 16 459 469 10.1016/j.copbio.2005.06.005 16005204

[B8] Nygren PA Skerra A Binding proteins from alternative scaffolds J Immunol Methods 2004 290 3 28 10.1016/j.jim.2004.04.006 15261569

[B9] Binz HK Amstutz P Pluckthun A Engineering novel binding proteins from nonimmunoglobulin domains Nat Biotechnol 2005 23 1257 1268 10.1038/nbt1127 16211069

[B10] Endo T Tamiya N Current view on the structure-function relationship of postsynaptic neurotoxins from snake venoms Pharmacol Ther 1987 34 403 451 10.1016/0163-7258(87)90002-7 3324114

[B11] Tsetlin V Snake venom α-neurotoxin and other 'three-finger' proteins Eur J Biochem 1999 264 281 286 10.1046/j.1432-1327.1999.00623.x 10491072

[B12] Miwa JM Ibanez-Tallon I Crabtree GW Sánchez R Sali A Role LW Heintz N Lynx1, an endogenous toxin-like modulator of nicotinic acetylcholine receptors in the mammalian CNS Neuron 1999 23 105 114 10.1016/S0896-6273(00)80757-6 10402197

[B13] Tsuji H Okamoto K Matsuzaka Y Iizuka H Tamiya G Inoko H SLURP-2, a novel member of the human Ly-6 superfamily that is up-regulated in psoriasis vulgaris Genomics 2003 81 26 33 10.1016/S0888-7543(02)00025-3 12573258

[B14] Fry BG Wüster W Kini RM Brusic V Khan A Venkataraman D Rooney AP Molecular evolution and phylogeny of elapid snake venom three-finger toxins J Mol Evol 2003 57 110 129 10.1007/s00239-003-2461-2 12962311

[B15] Teixeira-Clerc F Menez A Kessler P How do short neurotoxins bind to a muscular-type nicotinic acetylcholine receptor? J Biol Chem 2002 277 25741 25747 10.1074/jbc.M200534200 12006581

[B16] Bourne Y Talley TT Hansen SB Taylor P Marchot P Crystal structure of a Cbtx-AChBP complex reveals essential interactions between snake alpha-neurotoxins and nicotinic receptors EMBO J 2005 24 1512 1522 10.1038/sj.emboj.7600620 15791209 PMC1142565

[B17] Hirano T Akira S Taga T Kishimoto T Biological and clinical aspects of interleukin 6 Immunol Today 1990 11 443 449 10.1016/0167-5699(90)90173-7 2127356

[B18] Hirano T Kishimoto T Talal N Interleukin 6 and autoimmune diseases Molecular Autoimmunity 1991 Academic Press 177 194

[B19] Heinrich PC Behrmann I Haan S Hermanns HM Müller-Newen G Schaper F Principles of interleukin (IL)-6-type cytokine signalling and its regulation Biochem J 2003 374 1 20 10.1042/BJ20030407 12773095 PMC1223585

[B20] Maes M Yirmyia R Noraberg J Brene S Hibbeln J Perini G Kubera M Bob P Lerer B Maj M The inflammatory & neurodegenerative (I&ND) hypothesis of depression: leads for future research and new drug developments in depression Metab Brain Dis 2009 24 27 53 10.1007/s11011-008-9118-1 19085093

[B21] Rasmussen P Vedel JC Olesen J Adser H Pedersen MV Hart E Secher NH Pilegaard H humans IL-6 is released from the brain during and after exercise and paralleled by enhanced IL-6 mRNA expression in the hippocampus of mice Acta Physiol (Oxf) 2010 10.1111/j.1748-1716.2010.02223.x 21083649

[B22] Antil S Servent D Menez A Variability among the sites by which curaremimetic toxins bind to torpedo acetylcholine receptor, as revealed by identification of the functional residues of alpha-cobratoxin J Biol Chem 1999 274 34851 34858 10.1074/jbc.274.49.34851 10574958

[B23] Ferrari DM Soling HD The protein disulfide-isomerase family: unravelling a string of folds Biochem J 1999 339 1 10 10.1042/0264-6021:3390001 10085220 PMC1220120

[B24] Li J Zhang H Liu J Xu K Novel genes encoding six kinds of three-finger toxins in *Ophiophagus hannah *(king cobra) and function characterization of two recombinant long-chain neurotoxins Biochem J 2006 398 233 42 10.1042/BJ20060004 16689684 PMC1550305

[B25] Friguet B Chaffotte AF Djavadi-Ohaniance L Goldberg ME Measurements of the true affinity constant in solution of antigen-antibody complexes by enzyme-linked immunosorbent assay J Immunol Methods 1985 77 305 319 10.1016/0022-1759(85)90044-4 3981007

[B26] Hoogenboom HR Selecting and screening recombinant antibody libraries Nat Biotechnol 2005 23 1105 1116 10.1038/nbt1126 16151404

[B27] Braisted AC Wells JA Minimizing a binding domain from protein A Proc Natl Acad Sci USA 1996 93 5688 5692 10.1073/pnas.93.12.5688 8650153 PMC39121

[B28] Xu L Aha P Gu K Kuimelis RG Kurz M Lam T Lim AC Liu H Lohse PA Sun L Weng S Wagner RW Lipovsek D Directed evolution of high-affinity antibody mimics using mRNA display Chem Biol 2002 9 933 942 10.1016/S1074-5521(02)00187-4 12204693

[B29] Cho GS Szostak JW Directed evolution of ATP binding proteins from a zinc finger domain by using mRNA display Chem Biol 2006 13 139 147 10.1016/j.chembiol.2005.10.015 16492562

[B30] Beste G Schmidt FS Stibora T Skerra A Small antibody-like proteins with prescribed ligand specificities derived from the lipocalin fold Proc Natl Acad Sci USA 1999 96 1898 1903 10.1073/pnas.96.5.1898 10051566 PMC26708

[B31] Binz HK Stumpp MT Forrer P Amstutz P Plückthun A Designing repeat proteins: Well-expressed, soluble and stable proteins from combinatorial libraries of consensus ankyrin repeat proteins J Mol Biol 2003 332 489 503 10.1016/S0022-2836(03)00896-9 12948497

[B32] Xie MH Yuan J Adams C Gurney A Direct demonstration of MuSK involvement in acetylcholine receptor clustering through identification of agonist scFv Nat Biotechnol 1997 15 768 771 10.1038/nbt0897-768 9255792

[B33] Rowley MJ O'Connor K Wijeyewickrema L Phage display for epitope determination: a paradigm for identifying receptor-ligand interactions Biotechnol Annu Rev 2004 10 151 188 full_text 15504706 10.1016/S1387-2656(04)10006-9

[B34] Frederickson S Renshaw MW Lin B Smith LM Calveley P Springhorn JP Johnson K Wang Y Su X Shen Y Bowdish KS A rationally designed agonist antibody fragment that functionally mimics thrombopoietin Proc Natl Acad Sci USA 2006 103 14307 14312 10.1073/pnas.0602658103 16973749 PMC1599960

[B35] Cwirla SE Balasubramanian P Duffin DJ Wagstrom CR Gates CM Singer SC Davis AM Tansik RL Mattheakis LC Boytos CM Schatz PJ Baccanari DP Wrighton NC Barrett RW Dower WJ Peptide agonist of the thrombopoietin receptor as potent as the natural cytokine Science 1997 276 1696 1699 10.1126/science.276.5319.1696 9180079

[B36] McConnell SJ Dinh T Le MH Brown SJ Becherer K Blumeyer K Kautzer C Axelrod F Spinella DG Isolation of erythropoietin receptor agonist peptides using evolved phage libraries Biol Chem 1998 379 1279 1286 10.1515/bchm.1998.379.10.1279 9820589

[B37] Baneyx F Mujacic M Recombinant protein folding and misfolding in *Escherichia coli* Nat Biotechnol 2004 22 1399 1408 10.1038/nbt1029 15529165

[B38] Levandoski MM Caffery PM Rogowski RS Lin Y Shi QL Hawrot E Recombinant expression of alpha-bungarotoxin in *Pichia pastoris *facilitates identification of mutant toxins engineered to recognize neuronal nicotinic acetylcholine receptors J Neurochem 2000 74 1279 1289 10.1046/j.1471-4159.2000.741279.x 10693962

[B39] Love RA Stroud RM The crystal structure of alpha-bungarotoxin at 2.5 Å resolution: relation to solution structure and binding to acetylcholine receptor Protein Eng 1986 1 37 46 10.1093/protein/1.1.37 3507686

[B40] Tsernoglou D Petsko GA The crystal structure of a post-synaptic neurotoxin from sea snake at 2.2 Å resolution FEBS Lett 1976 68 1 4 10.1016/0014-5793(76)80390-0 964372

[B41] Silverman J Liu Q Bakker A To W Duguay A Alba BM Smith R Rivas A Li P Le H Whitehorn E Moore KW Swimmer C Perlroth V Vogt M Kolkman J Stemmer WP Multivalent avimer proteins evolved by exon shuffling of a family of human receptor domains Nat Biotechnol 2005 23 1556 1561 10.1038/nbt1166 16299519

[B42] Qiu XQ Wang H Cai B Wang LL Yue ST Small antibody mimetics comprising two complementarity-determining regions and a framework region for tumor targeting Nat Biotechnol 2007 25 921 929 10.1038/nbt1320 17676038

[B43] Atreya R Mudter J Finotto S Müllberg J Jostock T Wirtz S Schütz M Bartsch B Holtmann M Becker C Strand D Czaja J Schlaak JF Lehr HA Autschbach F Schürmann G Nishimoto N Yoshizaki K Ito H Kishimoto T Galle PR Rose-John S Neurath MF Blockade of interleukin 6 trans signaling suppresses T-cell resistance against apoptosis in chronic intestinal inflammation: evidence in crohn disease and experimental colitis in vivo Nat Med 2000 6 583 588 10.1038/75068 10802717

[B44] Wallenius V Wallenius K Ahrén B Rudling M Carlsten H Dickson SL Ohlsson C Jansson JO Interleukin-6-deficient mice develop mature-onset obesity Nat Med 2002 8 75 79 10.1038/nm0102-75 11786910

[B45] Chida D Osaka T Hashimoto O Iwakura Y Combined interleukin-6 and interleukin-1 deficiency causes obesity in young mice Diabetes 2006 55 971 977 10.2337/diabetes.55.04.06.db05-1250 16567518

[B46] Fee D Grzybicki D Dobbs M Ihyer S Clotfelter J Macvilay S Hart MN Sandor M Fabry Z Interleukin 6 promotes vasculogenesis of murine brain microvessel endothelial cells Cytokine 2000 12 655 665 10.1006/cyto.1999.0599 10843741

[B47] Woiciechowsky C Schöning B Cobanov J Lanksch WR Volk HD Döcke WD Early IL-6 plasma concentrations correlate with severity of brain injury and pneumonia in brain-injured patients J Trauma 2002 52 339 45 10.1097/00005373-200202000-00021 11834998

[B48] Quintana A Molinero A Borup R Nielsen FC Campbell IL Penkowa M Hidalgo J Effect of astrocyte-targeted production of IL-6 on traumatic brain injury and its impact on the cortical transcriptome Dev Neurobiol 2008 68 195 208 10.1002/dneu.20584 18000830

[B49] Nishimoto N Kishimoto T Yoshizaki K Anti-interleukin 6 receptor antibody treatment in rheumatic disease Ann Rheum Dis 2000 59 21 27 10.1136/ard.59.suppl_1.i21 PMC1766618 11053081

[B50] Su JL Lai KP Chen CA Yang CY Chen PS Chang CC Chou CH Hu CL Kuo ML Hsieh CY Wei LH A novel peptide specifically binding to interleukin-6 receptor (gp80) inhibits angiogenesis and tumor growth Cancer Res 2005 65 4827 4835 10.1158/0008-5472.CAN-05-0188 15930303

[B51] Feng J Li Y Shen B The design of antagonist peptide of hIL-6 based on the binding epitope of hIL-6 by computer-aided molecular modeling Peptides 2004 25 1123 1131 10.1016/j.peptides.2004.04.009 15245871

[B52] Martin F Toniatti C Salvati AL Venturini S Ciliberto G Cortese R Sollazzo M The affinity-selection of a minibody polypeptide inhibitor of human interleukin-6 EMBO J 1994 13 5303 5309 7957096 10.1002/j.1460-2075.1994.tb06864.x PMC395486

[B53] Zahnd C Wyler E Schwenk JM Steiner D Lawrence MC McKern NM Pecorari F Ward CW Joos TO Plückthun A A designed ankyrin repeat protein evolved to picomolar affinity to Her2 J Mol Biol 2007 369 1015 1028 10.1016/j.jmb.2007.03.028 17466328

[B54] Boder ET Midelfort KS Wittrup KD Directed evolution of antibody fragments with monovalent femtomolar antigen-binding affinity Proc Natl Acad Sci USA 2000 97 10701 10705 10.1073/pnas.170297297 10984501 PMC27086

[B55] Wrighton NC Balasubramanian P Barbone FP Kashyap AK Farrell FX Jolliffe LK Barrett RW Dower WJ Increased potency of an erythropoietin peptide mimetic through covalent dimerization Nat Biotechnol 1997 15 1261 1265 10.1038/nbt1197-1261 9359108

[B56] Johnson DL Farrell FX Barbone FP McMahon FJ Tullai J Kroon D Freedy J Zivin RA Mulcahy LS Jolliffe LK Amino-terminal dimerization of an erythropoietin mimetic peptide results in increased erythropoietic activity Chem Biol 1997 4 939 950 10.1016/S1074-5521(97)90302-1 9427659

[B57] Kubetzko S Balic E Waibel R Zangemeister-Wittke U Pluckthun A PEGylation and multimerization of the anti-p185HER-2 single chain Fv fragment 4D5: effects on tumor targeting J Biol Chem 2006 281 35186 35201 10.1074/jbc.M604127200 16963450

[B58] Bock GH Long CA Riley ML White JD Kurman CC Fleisher TA Tsokos M Brown M Serbousek D Schwietermann WD Nelson DL Characterization of a new IL-6 dependent human B-lymphoma cell line in long term culture Cytokine 1993 5 480 489 10.1016/1043-4666(93)90039-8 8142604

