# Supplementary figures and images for: Directed evolution of a three-finger neurotoxin by using cDNA display yields antagonists as well as agonists of interleukin-6 receptor signaling
Source: Mol Brain. 2011 Jan 7;4:2. doi: 10.1186/1756-6606-4-2 (PMC3024951; doi:10.1186/1756-6606-4-2)

**A**

LVCY**TNVLEPP**PGTLETCPDDFTCV**KKWEGGRRV**TQYCSHACAIP**ASYEFVH**CCQTDKCNG

**B**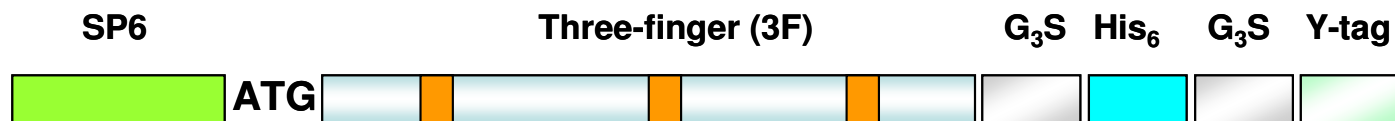**C**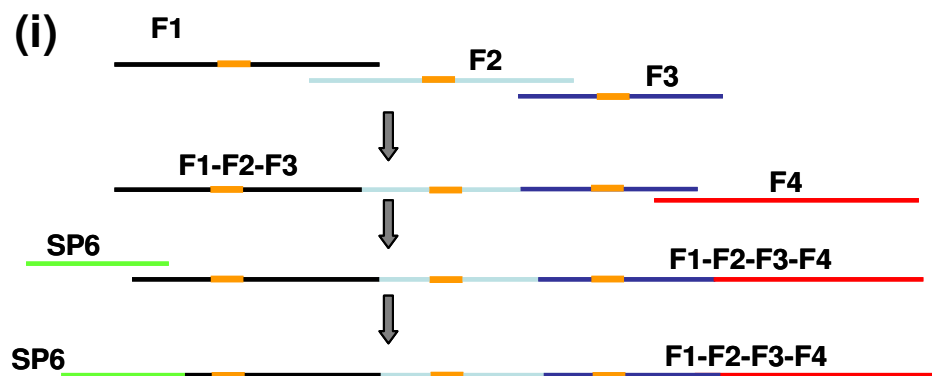**(ii)**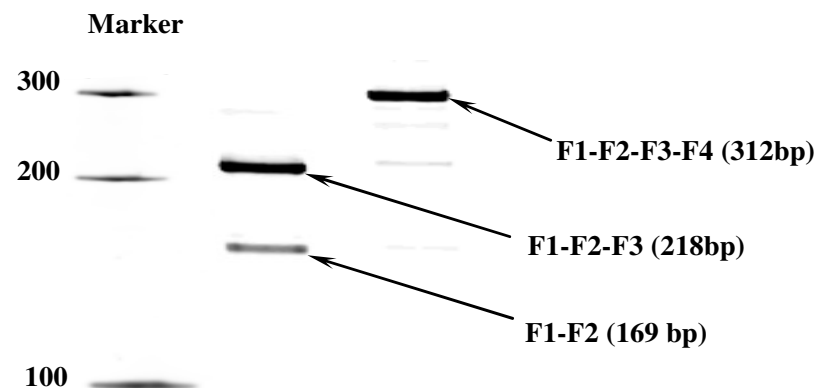**D**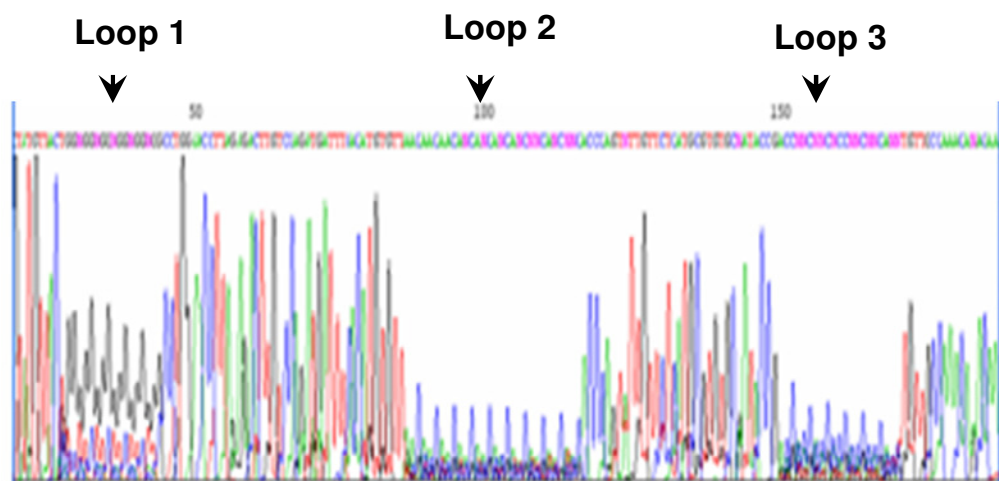

Supplement: Additional file 1 — Construction of the three-finger library. (A) Primary structure of the three-finger protein, MicTx3, used in this study. The four disulfide bonds formed between C1-C3, C2-C4, C5-C6 and C7-C8 are conserved among the three-finger proteins across a wide variety of species. The loops are shown in color and were randomized to generate the libraries. (B) Genetic construct adopted for cDNA display. SP6 contains the sp6 promoter, capping site and Xenopus globin untranslated sequence (UTR). ATG is the translation initiation codon. The 3F gene with the three randomized loops provides the basis of the library. G3S is spacer, 6XHis facilitates affinity purification by Ni-NTA, and Y-tag facilitates ligation of mRNA to the puromycin linker which contains the complementary Y-tag sequence. (C) Construction of library by overlap PCR. (i) The library was constructed by joining the fragments listed in supplementary Table-1 using overlap PCR in various steps. The portions marked in orange denote the randomized residues. (ii) Analysis of the PCR products by electrophoresis. The bands denote the respective fragments. (D) Direct sequencing of the PCR products of the library. The portions shown by arrows represent loops that were randomized. The peaks are smaller than normal due to the presence of a mixture of residues. [file 1756-6606-4-2-S1.PDF]

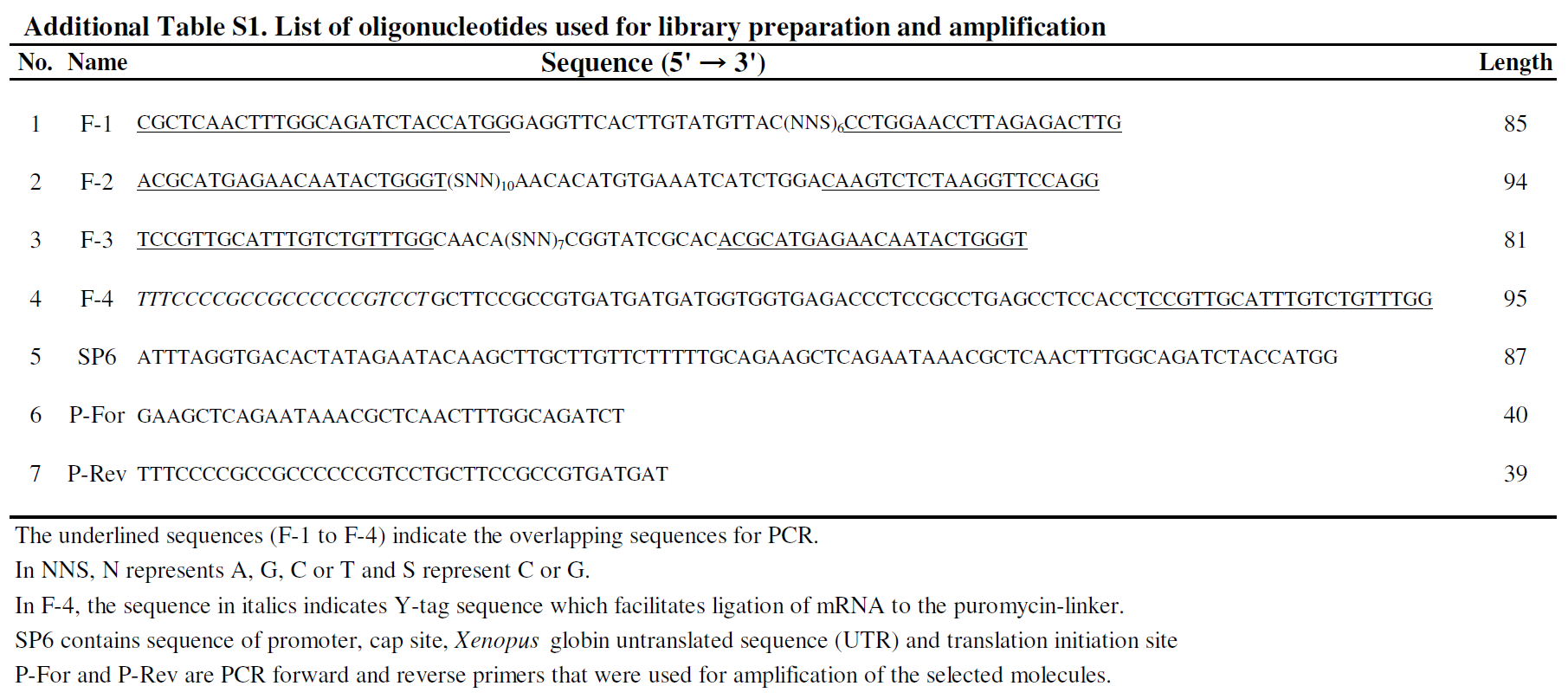

Supplement: Additional file 2 — List of oligonucleotides used for library preparation and amplification. Oligonucleotides that were used in the preparation of 3F library and the primers required for amplification of the selected library are listed. [file 1756-6606-4-2-S2.PNG]

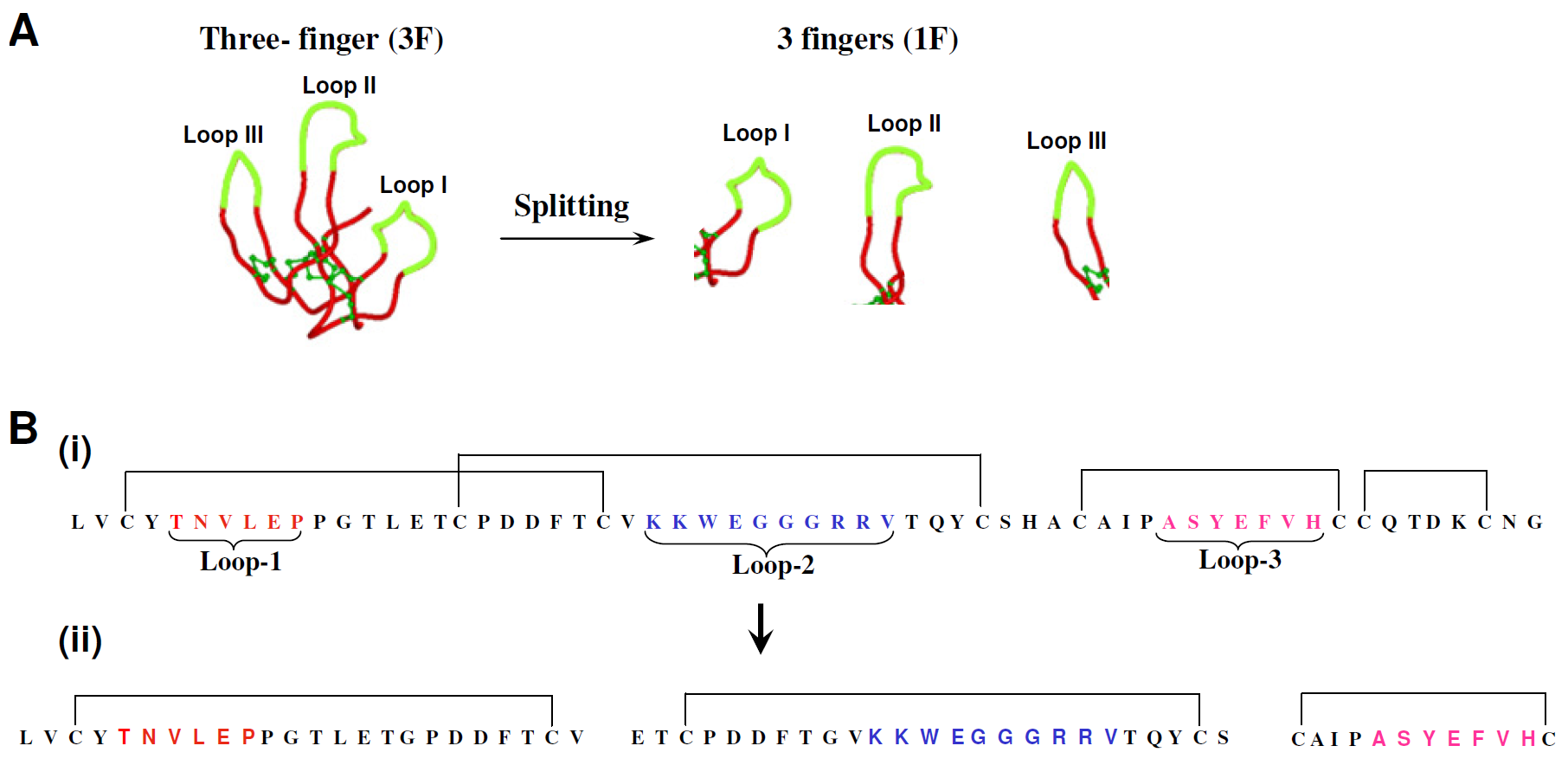

Supplement: Additional file 3 — Design of shorter peptides containing disulfide bonds from the parent 3F. (A) Scheme for designing peptides. 3F is a leaf-like flat molecule with the three fingers extending from the globular head. The three fingers were designed to split into three individual fingers. (B) Design of the peptides. Primary sequence of the three-finger protein. The four disulfide bonds are formed between C1-C3, C2-C4, C5-C6 and C7-C8. The loops are located between the disulfide bonds. (ii) Short peptides from the 3F sequence. The sequences of each of the peptides correspond to one of the loops. In Loop-1 (L-1), C2 was replaced by G, and in L-2, C3 was changed to G to restore the disulfide bonds as in the parent 3F. In the case of R10-14, C5 was found to be mutated to Y, however, a cysteine residue was selected in the randomized loop. Therefore, a disulfide bond was formed between this cysteine and C6. [file 1756-6606-4-2-S3.PNG]
